# Rapid photocatalytic dye degradation, enhanced antibacterial and antifungal activities of silver stacked zinc oxide garnished on carbon nanotubes

**DOI:** 10.1038/s41598-024-64746-6

**Published:** 2024-06-18

**Authors:** Snehal S. Wagh, Akanksha S. Chougale, Avinash A. Survase, Rajendra S. Patil, Nithesh Naik, Mu. Naushad, Habib M. Pathan

**Affiliations:** 1 Department of Polytechnic, Dr. Vishwanath Karad MIT World Peace University, Pune, Maharashtra 411038 India; 2 PSGVPM’s ASC College, Shahada, Maharashtra 425409 India; 3 Advanced Physics Laboratory, Department of Physics, Savitribai Phule Pune University, Pune, Maharashtra 411007 India; 4 Department of Microbiology, Rayat Institute of Research and Development, Satara, Maharashtra 415001 India; 5 Department of Mechanical and Industrial Engineering, Manipal Institute of Technology, Manipal Academy of Higher Education, Manipal, Karnataka 576104 India; 6 Department of Chemistry, College of Science, King Saud University, P.O. Box 2455, Riyadh, 11451 Saudi Arabia

**Keywords:** Silver-doped zinc oxide, CNT, Photocatalysis, Antibacterial, Antifungal, Nanoscience and technology, Physics

## Abstract

A composite of Zinc oxide loaded with 5-weight % silver decorated on carbon nanotubes (Ag-loaded ZnO: CNT) was synthesized using a simple refluxed chemical method. The influence of deviation in the weight % of carbon nanotube loading on photocatalytic dye degradation (methylene blue and rose bengal) and antibiotic (antimicrobial and antifungal) performance was investigated in this study. The light capture ability of Ag-loaded ZnO:CNT in the visible region was higher in photocatalytic activity than that of Ag-loaded ZnO and ZnO:CNT. The bandgap of the Ag-loaded ZnO: CNT was tuned owing to the surface plasmon resonance effect. The photocatalytic degradation investigations were optimized by varying the wt% in CNTs, pH of dye solution, concentration of the dye solution, and amount of catalytic dose. Around 100% photocatalytic efficiency in 2 min against MB dye was observed for Ag doped ZnO with 10 wt% CNT composite at pH 9, at a rate constant 1.48 min^−1^. *Bipolaris sorokiniana* fungus was first time tested against a composite material, which demonstrated optimum fungal inhibition efficiency of 48%. They were also tested against the bacterial strains *Staphylococcus aureus*, *Bacillus cerius*, *Proteus vulgaris*, and *Salmonella typhimurium*, which showed promising antibacterial activity compared to commercially available drugs. The composite of Ag doped ZnO with 5 wt% CNT has shown competitive zone inhibition efficacy of 21.66 ± 0.57, 15.66 ± 0.57, 13.66 ± 0.57 against bacterial strains *Bacillus cerius*, *Proteus vulgaris*, and *Salmonella typhimurium* which were tested for the first time against Ag-loaded ZnO:CNT.

## Introduction

The textile industrial zone is thought to be the dominant dye sector and a source of wastewater. The unrestricted use of industrial wastewater contains a variety of chemicals that are damaging to the environment and community health. Substantial volumes of water and dyes are used in broad processing, such as printing and dyeing textiles. They discharge a large amount of sewage, and many of them contain colors that are harmful to the environment and community. Synthetic dyes and other organic pollutants are often used in various industries, including textiles, pulp, letterpress, leather, body cosmetics, plastic, and food^1^. As a result, eliminating them is vital and challenging. As these dyes separate into aromatic structures, they have lethal, mutagenic, and oncogenic potentials that affect their lives. Moreover, dyes can prevent light from inflowing into aquatic environments and impair aquatic life^2^. It is vital to confiscate the colors of sewage and discarded water to maintain the network. Methylene blue (MB), which is sometimes referred to as methyl thioninium chloride, and rose bengal (RB) are representative pollutants. They are cationic organic dyes composed of synthetic compounds with strong odors. It is poisonous to all living organisms and causes cancer. It affects the respiratory system and irritates the skin and eyes^3,4^. It is known that drinking water contains a substantial amount of traces of MB dye, which causes subcutaneous tissue-borne sarcoma in both humans and animals. Dyes have long-lasting effects on aquatic life, especially plants, as they reduce natural purification and photocatalysis by obstructing light diffusion. As a result, the dye must be seized before being released into freshwater^5^. Owing to its distinctive optical and electrical characteristics, zinc oxide (ZnO), a well-known semiconductor material with a broadband gap (3.37 eV and n-type conductivity, has gained popularity as a photocatalyst. ZnO is a viable option for several applications due to its photocatalytic activity, modest toxicity, strong stability, and affordability^6,7^. The narrow spectrum range of ZnO, resulting from its wide band gap and high charge carrier recombination rate, limits its photocatalytic efficacy^8^. As a result, the accumulation of a substantial amount of ZnO is a suitable technique. When an element is added, it can serve as a place to supply electrons or soothe catalysts to materials with a large surface area, which will help with electron transmission in the final composite^9–11^. Sher et.al developed g–C_3_N_4_–ZnO composite with Mo, Ag and Sn doping as a promising candiadate to degrade water moieties and bacterial inhibition^11–13^. In our previous study, Ag-loaded ZnO was successfully synthesized for use in clean wastewater. The amalgamation of Ag with ZnO helped in tuning the band gap and prolonged the recombination time of the charge carriers to extend the degradation rate. Since their invention, carbon nanotubes (CNTs) have drawn attention worldwide. Depending on the helicity and tube diameter, they can be semiconducting, semi-metallic, or metallic. The notable properties of multi-walled carbon nanotubes (MWCNTs) include a high aspect ratio, large surface area, strong adsorptive ability, good catalytic activity, outstanding mechanical strength, elasticity, and good electrical and thermal conductivity^14^. It was assumed that these were sheets of coiled graphene. Because carbon nanotubes (CNTs) are incorporated into host materials to change or improve their properties, CNT-based composites have attracted attention. Owing to their non-polar nature, CNTs are hydrophobic. The π electrons outside the carbon nanotubes create an extremely strong connection. The functionalization process can change the hydrophobic nature of CNTs. The process of attaching individual atoms or molecules to carbon nanotubes is known as the functionalization. Functionalized multi-walled carbon nanotubes (MWCNTs) are widely employed in catalyst synthesis. According to Zeo et al.^15^, CNT-loaded ZnO blends perform better than pure ZnO in preventing dye degradation. A ZnO:CNT composite was created using a thermal technique to break down carcinogenic acetaldehyde for 60 min while being exposed to laser radiation^16^. To break down methylene blue, ZnO/N-CNTs were created via a chemical precipitation method, and benzoic acid produced better outcomes^17^. ZnO/Co–Ni–Al layered CNT composite decomposed the acid red at pH 5.5 and demonstrated an efficiency of 96.2% when exposed to visible light for 120 min^18^. Hence, CNT’s are promising candidates for photocatalytic degradation of hazardous pollutants^19^.

In this study, the antifungal and antimicrobial characteristics of Ag-loaded ZnO:CNT were explored. Functionalized multi-walled carbon nanotubes have recently been demonstrated to reduce immunological discomfort in mice and macrophages while also improving colloidal characteristics without compromising their distinctive antibiosis efficacy^20^. An extensive study of antifungal and antimicrobial agents is required to elucidate the promising characteristics of Ag-loaded ZnO:CNT; hence, following the literature, it was discovered that surface changes in MWCNTs were more effective against Fusarium graminearum^21^. The cell membrane, which defends bacteria from the severity of the environment, undergoes physicochemical changes resulting in inactivation by the composite material^22^. When combined with toyocamycin, CNT demonstrated specific toxicity against Candida albicans^23^. Bacterial degradation is commonly associated with oxidative stress caused by ROS generated by functional materials. Spot blotch infection in wheat is triggered by the fungus, *Bipolaris sorokiniana*. This disease can seriously harm crops and the economy^24^. Therefore, CNTs are a promising option for the prevention of fungal and bacterial development^19^. The water quality is lowered by organic toxic waste and a few other active poisons found in wastewater. The toxins are essentially bits and pieces of bacteria, which cause the pathogenicity of water to increase^25,26^. With band gap energies ranging from 1.0 to 3.8 eV, numerous metal oxide semiconductor materials are engaged as heterogeneous semiconductor photocatalysts as well as antifungal and antimicrobial-resistant materials^27,28^. Visible-range band-gap semiconducting materials are critical photocatalysts and antibacterial agents because it is important to use sunlight efficiently to produce energy, sanitize water, and ensure ecological safety. To understand its influence on variations in color, dye concentration, catalytic dose, and pH of the dye solution, we synthesized functionalized carbon nanotubes decorated with Ag-loaded ZnO. The antibacterial effect of Ag-loaded ZnO:CNT was tested against the bacterial strains *Staphylococcus aureus* (NCIM-2654), *Bacillus cerius* (NCIM-2703), *Proteus vulgaris* (NCIM-2813), and *Salmonella typhimurium* (NCIM-2501). Additionally, we are the first to demonstrate antifungal action on a wheat spot blotch utilizing Ag-loaded ZnO:CNT composites.

## Experimental section

### Material

In current efforts, analytical-grade compounds have been used in the due course of investigation. Zinc acetate dihydrate (Sisco Research Lab (SRL)), diethylene glycol (Sisco Research Lab (SRL)), silver nitrate (Thomas Baker (Chemicals) Pvt. Ltd.), purity 99.8%, sodium hydroxide pellets (ACROS organics), multi-walled carbon Nano Tube (Monad Nano Tech Pvt. Ltd, India), methylene blue (Sisco Research Lab (SRL)), rose bengal (Molychem), and potato dextrose agar medium (Himedia, Mumbai, India) were commercially purchased. All solutions used in this investigation were prepared using double-distilled water (DDW).

### Characterization

The crystal structure of the Ag-loaded ZnO crystal structure was examined by X-ray diffraction (XRD) (D/B max-2400, Rigaku, USA) to ascertain their phase and average crystallite size. The morphology was examined using a JEOL JSM 6360-A field-emission scanning electron microscope (FESEM). Optical analysis was conducted using a UV-visible spectrophotometer (JASCO V-670, Germany), photoluminescence (PL) was observed and recorded using a PL spectrometer (Horiba Fluorolog), the X-ray photoelectron spectroscopy (XPS) was executed on PHI Versaprobe III by using Al Ka X-rays. An OSRAM 300 W halogen lamp was used as the visible light source (emission range: 400–800 nm) for the photocatalytic degradation of the MB and RB dyes.

### Preparation of silver loaded zinc oxide: carbon nano tube composites

Initially, we functionalized the already purchased carbon nanotubes by adding sodium lauryl sulfate (SLS) at a ratio of 1:20. The SLS plays quite an essential role in the functionalization of CNTs, upon functionalization of CNTs, the attraction of their surfaces to several solvents and polymer increases. The imperfections on the CNTs shaped by SLS were alleviated by attachment with different functional group formations, as described in our previous work^29,30^. Furthermore, Ag-loaded ZnO:CNT nanoparticles with (5, 10, 15, and 20) wt% functionalized CNTs was prepared by adding the desired amount of zinc acetate and silver nitrate in double distilled water. The reflux chemical method was implemented in which 1 M zinc acetate and 5 wt% silver nitrate was added to 150 ml distilled water at 80 °C for 2 h. using 20 ml ethylene glycol. This solution was mixed with functionalized CNTs under constant stirring for 2 h at 80 °C using the refluxed chemical method. A separate 2 M sodium hydroxide (NaOH) solution was prepared in 150 ml DDW and poured slowly at a rate of 2 drops per second. to the above solution. The solution was agitated by stirring for 3 h. Consequently, a blackish solution was obtained. The blackish solution was cooled overnight, rinsed, and washed multiple times with ethyl alcohol. The precipitate was then collected and incubated at 70 °C for 24 h. This precipitate was crushed into a fine powder, which was then annealed at 300 °C for approximately 3 h. Thus, Ag-loaded ZnO/CNT nanoparticles were prepared. These Ag-loaded ZnO/CNT samples were labeled as 5CNT, 10CNT, 15CNT, and 20CNT respectively for (5, 10, 15, and 20) wt% of CNT’s.

### Antifungal activity measurement

The antifungal activity of the silver-loaded zinc oxide: carbon nanotube was evaluated using the agar diffusion process, as previously described^29^. To perform this test, potato dextrose agar (PDA) medium was mixed into 0.1 L of double distilled water and boiled until completely dissolved. The PDA and glass Petri dishes were autoclaved for 30 min at 15 Pa of pressure to sterilize them, and the PDA medium was spread into the Petri dishes using a laminar flow chamber. Following the solidification of the medium, 20 mg of the produced material was placed on the plate. The center of the Petri plate was then injected with a fungal disc from *Bipolaris sorokiniana*^29^. The potato dextrose agar disc was removed from the center using a sterile cork borer for inoculation. A control without Ag-loaded ZnO:CNT composites was used for comparison with incubation of the plates for 72 h at 25 °C. The growth of the fungal zone was also observed. We measured the diameter of such inhibitors to determine their antifungal activity using the poisoned food approach^31^.

### Antimicrobial activity measurement

During the water refinement process, several supplementary toxins and organic pollutants are present in sewage. These toxic materials contain bits and pieces of microorganisms, and it was previously discovered that organic metals are primarily responsible for the proliferation of dangerous bacteria in water^32^. Consequently, it was discovered that photocatalysts possess the potential to allow microbial pollutants to reside on them^33^. Examining the antibacterial activity of Ag-loaded ZnO:CNT (50 µg/mL) and commercial antibiotics (50 µg/mL), a notable zone of inhibition was found. Microbial cultures were cultivated in inocula using a sterile saline solution. Nutrient agar plates were used as substrates for the development of bacteria. *Staphylococcus aureus* was allowed to grow on sterile savored agar plates; similarly, *Bacillus cereus*, *Proteus vulgaris*, and *Salmonella typhimurium* cultures were allowed to thrive on clean nutrient agar plates. Powdered 5CNT, 10CNT, 15CNT, and 20CNT were mixed with sterile distilled water and dimethyl sulfoxide (DMSO) using a micropipette. To track the antibacterial activity, the plates were incubated for twenty-four hours at 37 °C^34^.

### Photodegradation activity

We generated dye solutions of various concentrations in DDW to evaluate the photocatalytic degradation activity, and the sample was investigated using a UV-visible study, which yielded the absorbance of an unadulterated dye solution of a given concentration. A fixed number of catalyst samples were combined with MB and RB dye solutions. The solutions were held in a gloomy environment for 30 min with regular agitation to avoid light interactions and to sustain adsorption/desorption symmetry among the photocatalyst surfaces and dye molecules^35^. The solutions were then exposed to visible light to evaluate their photocatalytic degradation ability. Small samples were taken at regular intervals for UV-Vis measurements until the total disintegration of the dye solution was observed. The fading of the characteristic absorbance peak from the UV-Vis experiments confirmed dye degradation. By pulling out aliquots for UV-Vis spectroscopic measurement at time intervals of 5 min for each sample, the absorbance maxima of MB and RB were observed at 664 nm and 546 nm, respectively.

## Results and discussion

### X-ray diffraction analysis

**Figure 1:**
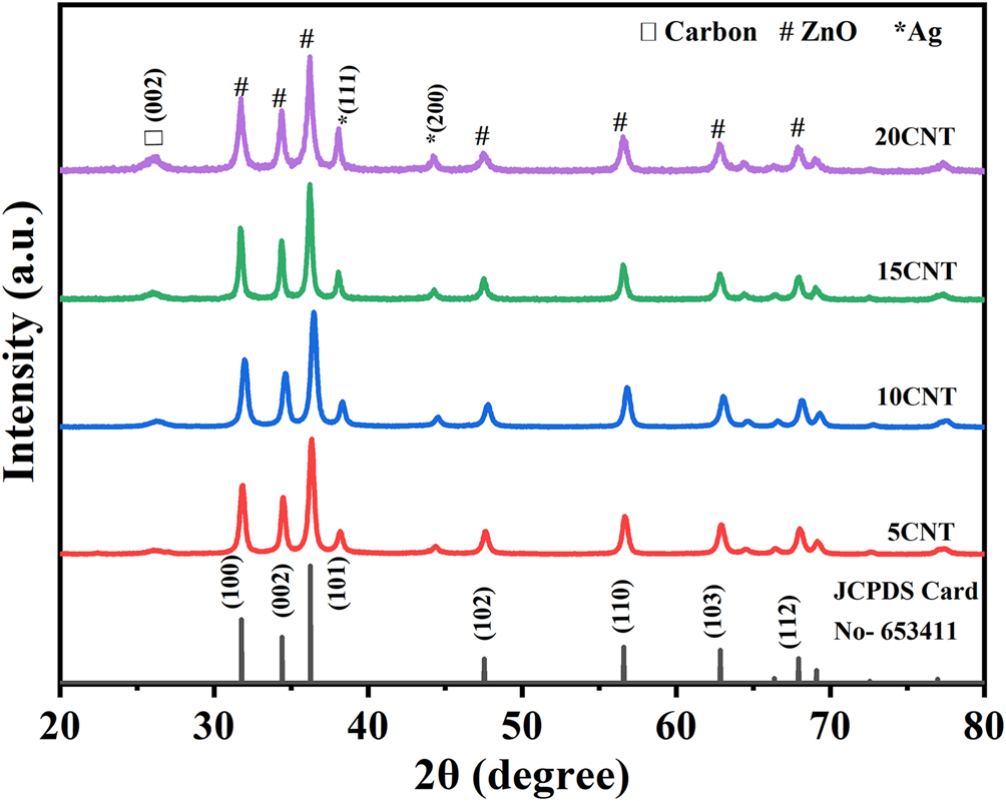
X-ray diffraction pattern of 5CNT, 10CNT, 15CNT, and 20CNT composites.

Figure 1 shows the X-ray diffraction pattern array of the 5 wt% Ag loaded ZnO composed with (5 to 20) wt% CNT’s. The major ZnO peaks have hexagonal phases, which is in good agreement with JCPDS Card No. 653411 for all samples (indicated by # mark in Fig. 1). As we have amalgamated Ag with ZnO, the (111) and (200) peaks (indicated by * mark in Fig. 1) corresponding to Ag are observed with low-intensity minor phase development, as reported in our previous work^36^. Silver was found in interstices as well as in substitutional Zn sites, as reported elsewhere. The existence of the (002) carbon peak was (indicated by □ mark in Fig. 1) also observed in all the samples owing to composite formation with CNTs. Other diffraction peaks show no significant change as a result of CNT integration, which is consistent with the physical interaction of CNT with the Ag-ZnO array^37^. The integration of CNTs into Ag-loaded ZnO precludes the secondary phase formation in the XRD configuration of the materials. The Scherrer equation was employed to calculate the microstrain, dislocation density, and average crystallite size for the (100), (002), (101), (102), (110), (103), (200), and (112) peaks of ZnO, as summarized in Table 1.

**Table 1:** Parameters calculated from XRD analysis.

Catalyst	Micro strain ‘ε’ (× 10^−3^)	Dislocation density ‘δ’ (m^−2^) (× 10^−3^)	Average crystallite size (D) (nm)
5CNT	2.07	2.15	21
10CNT	2.15	2.27	19
15CNT	1.61	1.32	25
20CNT	1.99	2.13	22

The microstrain and dislocation density were the highest for 10CNT, and the mean crystallite size was 19 nm for 10CNT. This could have been caused by the occurrence of silver ions in the ZnO array hindering the formation of crystal grains, and resistance to grain boundary growth was created, possibly as a result of the Zinner-Pinning effect^38^.

### Optical analysis

**Figure 2:**
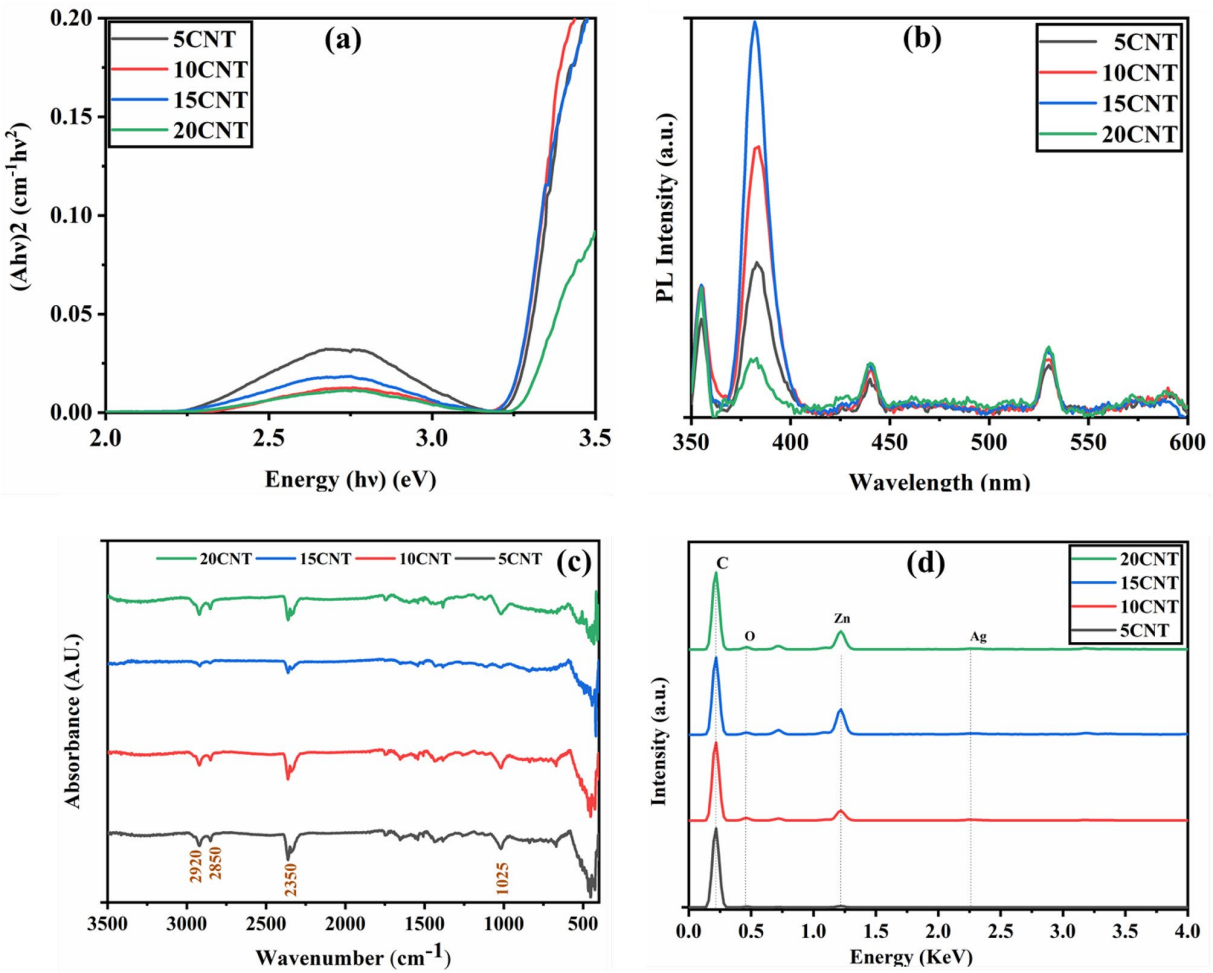
(**a**) Tauc plot of UV absorption spectra (**b**) Photoluminescence spectra (**c**) FTIR spectra and (**d**) EDS analysis of 5CNT, 10CNT, 15CNT, and 20CNT composites.

The absorption spectra of the Ag-loaded ZnO/CNT composites are shown in the inset of Fig. 2a. Owing to the relocation of charges from the valence band to the conduction band of ZnO, a strong absorption edge was observed in the UV region (200–420 nm)^6,39^. As shown in Fig. 2a, the Tauc plot was used to measure the bandgap energy (Eg) of the composites. The bandgap energies (Eg) of 5CNT, 10CNT, 15CNT, and 20CNT were found to be around 3.25 eV at the absorbance maxima obtained of 359 nm. The accumulation of silver ions produces substantial alterations in the absorption spectra of ZnO, causing high absorbance in the complete visible spectrum in the form of a bulge, owing to the surface plasmon band^40^. The increase in luminescence can be caused by surface plasmon scattering^4^. At the interface of Ag and ZnO in the array, the localized electric field associated with metal ions is amplified, inducing surface plasmon excitation in the Ag nanoparticles positioned at the interface, which enhances the visible absorption of light photons^41^. As a result, the existence of Ag nanoparticles on ZnO materials causes an increase in visible light capture due to the plasmon band on the Ag surface^42^.

The conduction band potential and valance band potential of Ag-ZnO were measured by using the Mulliken electronegativity theory^13^

1 $${\text{E}}_{{{\text{VB}}}} = {\text{X}} - {\text{E}}_{{\text{e}}} + 0.{\text{5E}}_{{\text{g}}}$$

2 $${\text{E}}_{{{\text{CB}}}} = {\text{E}}_{{{\text{VB}}}} - {\text{E}}_{{\text{g}}}$$

where, X is the absolute electronegativity of the semiconductor and for ZnO it is 5.76 eV (vs NHE), E_g_ is the band gap energy of the semiconductor, E_VB_ is the valence band edge potential of semiconductor and E_e_ is the energy of free electrons and it is ~ 4.5 eV (vs NHE) and E_CB_ shows the conduction band edge potential^43^. The calculated values of valence band and conduction band potentials were 2.88 eV (vs NHE) and − 0.37 eV (vs NHE) respectively. Additionally, the work functions of Ag and ZnO are 4.3 and 5.2 eV, respectively; thus, the transmission of an electron from Ag to ZnO is suitable, which is in good agreement with photoluminescence spectroscopy studies^44,45^. The work function of functionalized MWCNT is 5.1 eV^46^. Also, it is demonstrated that MWCNT turns to be photosensitizer by captivating light so that they get excited to higher energy level leading to electron transfer.

Photoluminescence spectroscopy is a broad technique used to investigate the reunion of light-induced charge carriers in semiconductors^47,48^. The simultaneous recombination of photogenerated electron–hole pairs and radiation produces PL spectra with small PL intensity, indicating a sluggish reunion rate^12^ Here, we recorded the PL spectra of all composite samples under ambient thermal conditions, as shown in Fig. 2b, when excited at 320 nm. The PL spectrum showed stronger absorption at 380 nm and poorer absorption at 354 nm in the UV region^49,50^. The emission characteristics of all samples are comparable, with the peak at approximately 530 nm showing lowered intensities due to the transfer of electrons from the Zn_i_ level positioned beneath the conduction band to the valence band, signifying quenching of PL emission^51^. The plasmonic absorption of Ag nanoparticles may be responsible for the decrease in the visual emission intensity of the catalysts^49,50,52,53^. UV radiation due to near-band emission can be initiated by the wide band gap of ZnO. Furthermore, a couple of frail bands in the visible area near 430–440 nm could be caused by surface flaws of ZnO nanoparticles and bound pairs of charge carriers. Hence, it extends its support to the notion of integration of Ag nanoparticles and CNTs, which increases the lifetime of photogenerated charge carriers and subsequently raises the photocatalytic activity owing to lowered surface deficiencies in the Ag-loaded ZnO:CNT composite structure.

FT-IR spectra depict information about a specific substance, functional groups, molecular shape, and inter-/intramolecular interactions. We employed the room-temperature KBr pellet method to study the FT-IR spectra of the 5CNT, 10CNT, 15CNT, and 20CNT nanocomposites, as depicted in Fig. 2c. The spectra were run over the wave number range of 400–4000 cm^−1^. The absorption band between 2850 and 2920 cm^−1^ was found to be an asymmetric stretching mode for the C-H bond, which may be caused by sodium lauryl sulfate residues. Figure 2d shows the EDS analysis of 5CNT, 10CNT, 15CNT, and 20CNT composites.

The absorption band at 2350 cm^−1^ was discovered to correspond to CO_2_. In addition, the H–O–H bending vibration of water was observed at 1530 cm^−1^, while the band near 1030 cm^−1^ represented the asymmetric stretching of C–O^54^. The vibration modes of Zn–O correspond to the bands in the low wavenumber area of 435 cm^−1^^55^. This implies that the hydroxyl and carboxylic groups present on the exterior of the catalysts improve photocatalytic activity^56^.

### Morphological analysis

#### FESEM and EDS studies

**Figure 3:**
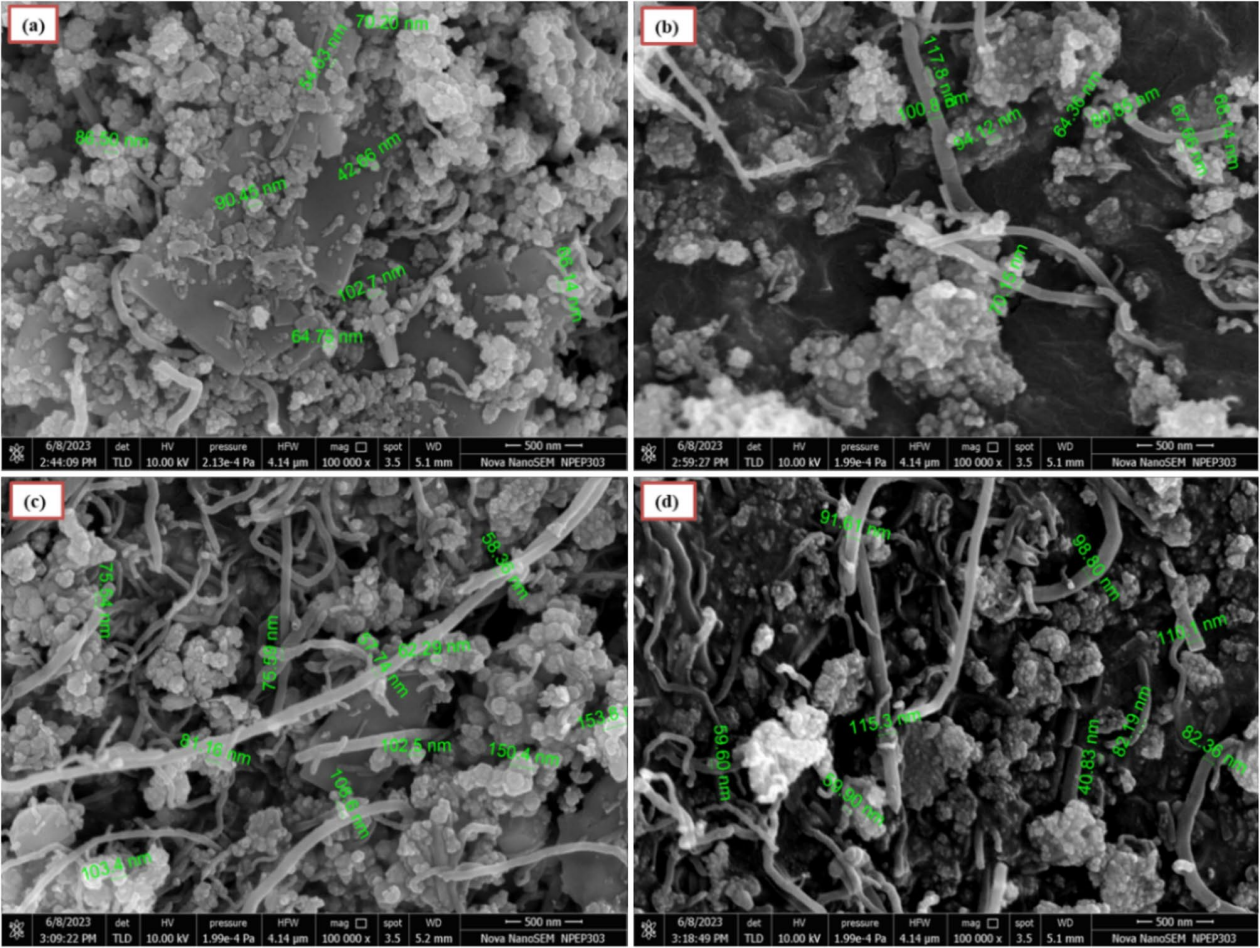
FESEM micrograph of (**a**) 5CNT (**b**) 10CNT (**c**) 15CNT (**d**) 20CNT composites.

FESEM images of the 5CNT, 10CNT, 15CNT, and 20CNT composites at 500 nm magnification are shown in Fig. 3. It is found that there is an agglomeration of Ag-loaded ZnO nanoparticles displaying cauliflower-like morphology. Each FESEM micrograph shows varying particle sizes ranging from 60 to 90 nm, which may be due to the change in the weight percentage contribution of CNT to the material. Larger particles occur owing to the agglomeration effect and the concentration of nucleation sites. The average particle size and diameter of the CNT calculated using the FESEM images were approximately 75 nm and 80 nm, respectively.

Figure 2d. shows the EDS spectrum for 5CNT, 10CNT, 15CNT and 20CNT. The composition and distribution of Ag-loaded ZnO:CNT composite nanoparticles are indicated by EDS mapping, which shows the even distribution of CNTs in the Ag Ag-loaded ZnO array. The spreading of the Zn and oxygen peaks in the EDS mapping confirms the purity of ZnO. The presence of Ag and carbon peaks in the Ag-loaded ZnO:CNT arrangement supplements the composite development in the matrix^57^.

#### X-ray photoelectron spectroscopy (XPS) studies

**Figure 4:**
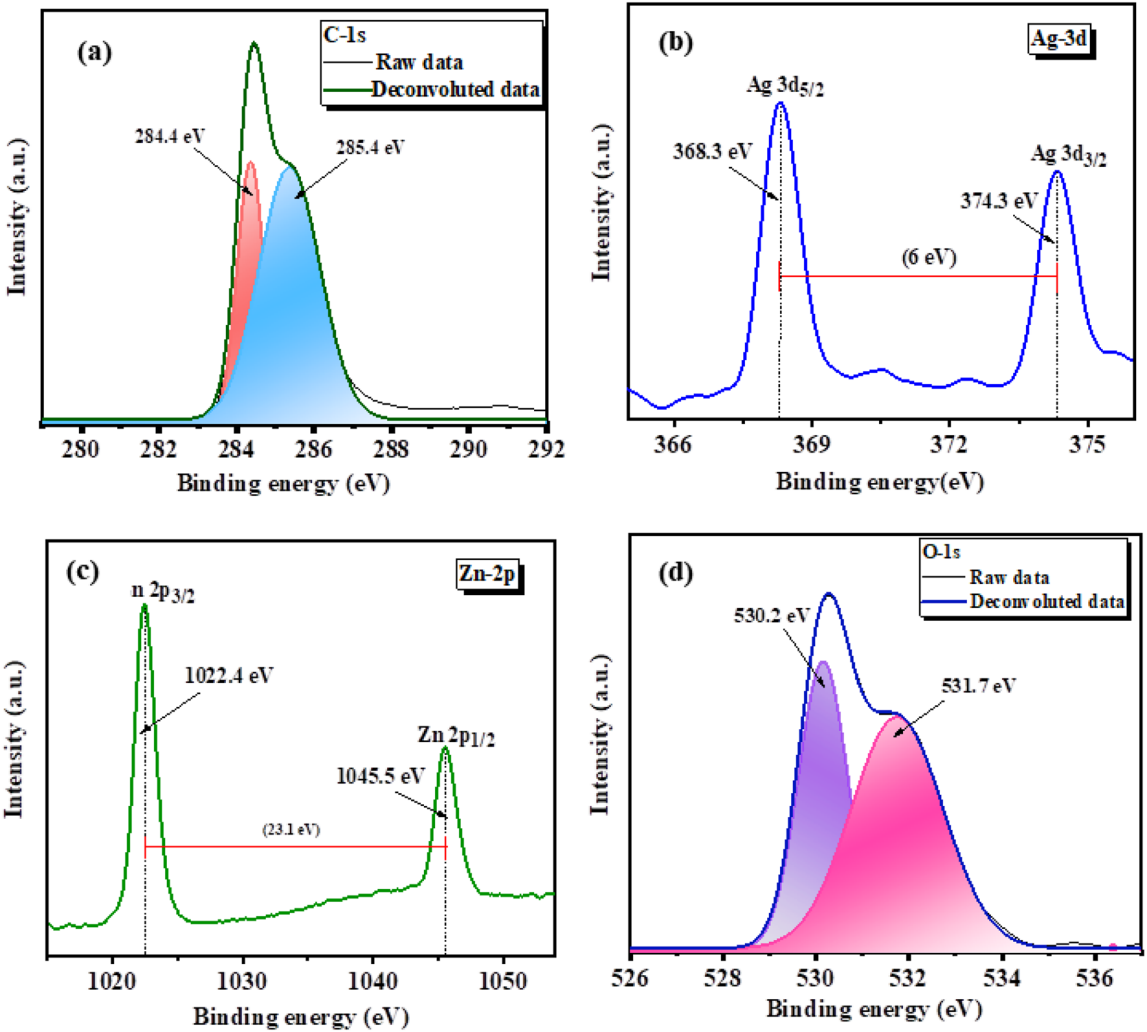
High resolution XPS of Ag doped ZnO:CNT nanocomposite.

Elemental composition and oxidation states of Ag doped ZnO/(10%)CNT were studied with the help of x-ray photoelectron spectroscopy (Fig. 4). As shown in Fig. 4a, the C 1s spectrum shows two peaks observed at binding energies of 284.4, and 285.4 eV that can link to C=C, and C–C respectively^58,59^. The peak at 284.4 is due to sp^2^ hybridized carbon atoms whereas different functional groups such as carboxyl and hydroxyl are attached to the surface of CNT are responsible for the peak at higher binding energy of 285.4 correspond to the sp^3^ hybridized carbon atoms^60^. Moreover, Ag 3d spin–orbit coupling is apparent from Fig. 4b, where two discrete peaks observed at binding energies 368.3 and 374.3 eV accredited to Ag 3d_5/2_ and Ag 3d_3/2_, respectively. Both peaks have 6 eV difference in their binding energies approves the presence of the Ag in the metallic nature over the surface of the ZnO^61^. Zn with 2^+^ valance state in ZnO is confirmed with Fig. 4c displaying the two binding energy peaks of Zn-2p_3/2_ and Zn-2p_1/2_ with energy difference of 23.1 eV corresponding to spin–orbit doublet. Also the O1s spectrum shown two peaks at 530.2 and 531.7 eV (Fig. 4d). The peak at 530.2 eV was due to O_2_^-^ ions attached with zinc in ZnO crystal structure, and the peak at 531.7 can be attributed to oxygen deficiencies or presence of hydroxyl and carboxyl group due to surface defects^62^.

### Photocatalytic studies

**Figure 5:**
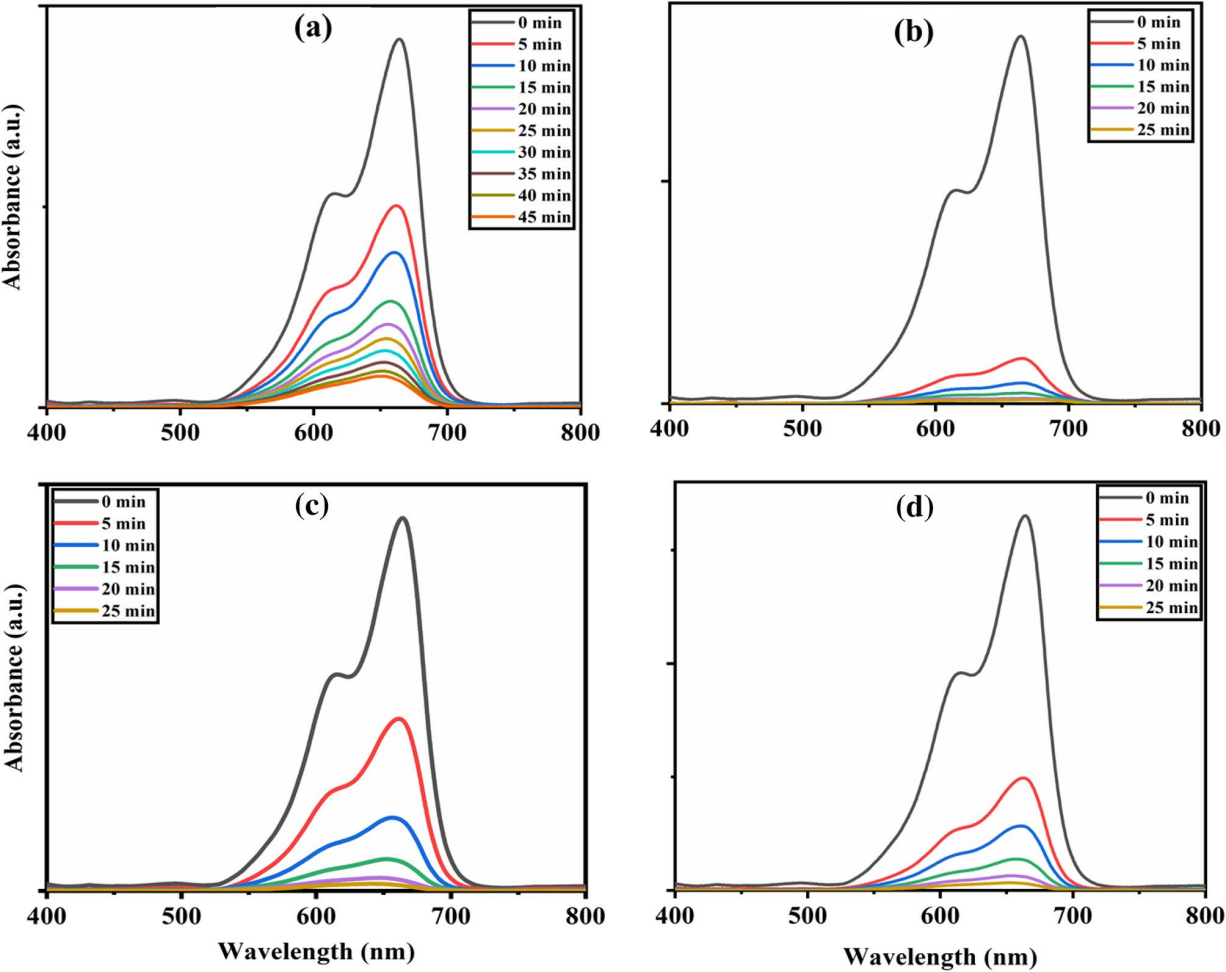
Photocatalytic degradation of (**a**) 5CNT (**b**) 10CNT (**c**) 15CNT (**d**) 20CNT composites against as prepared 10 ppm MB dye solution under visible light irradiation.

**Figure 6:**
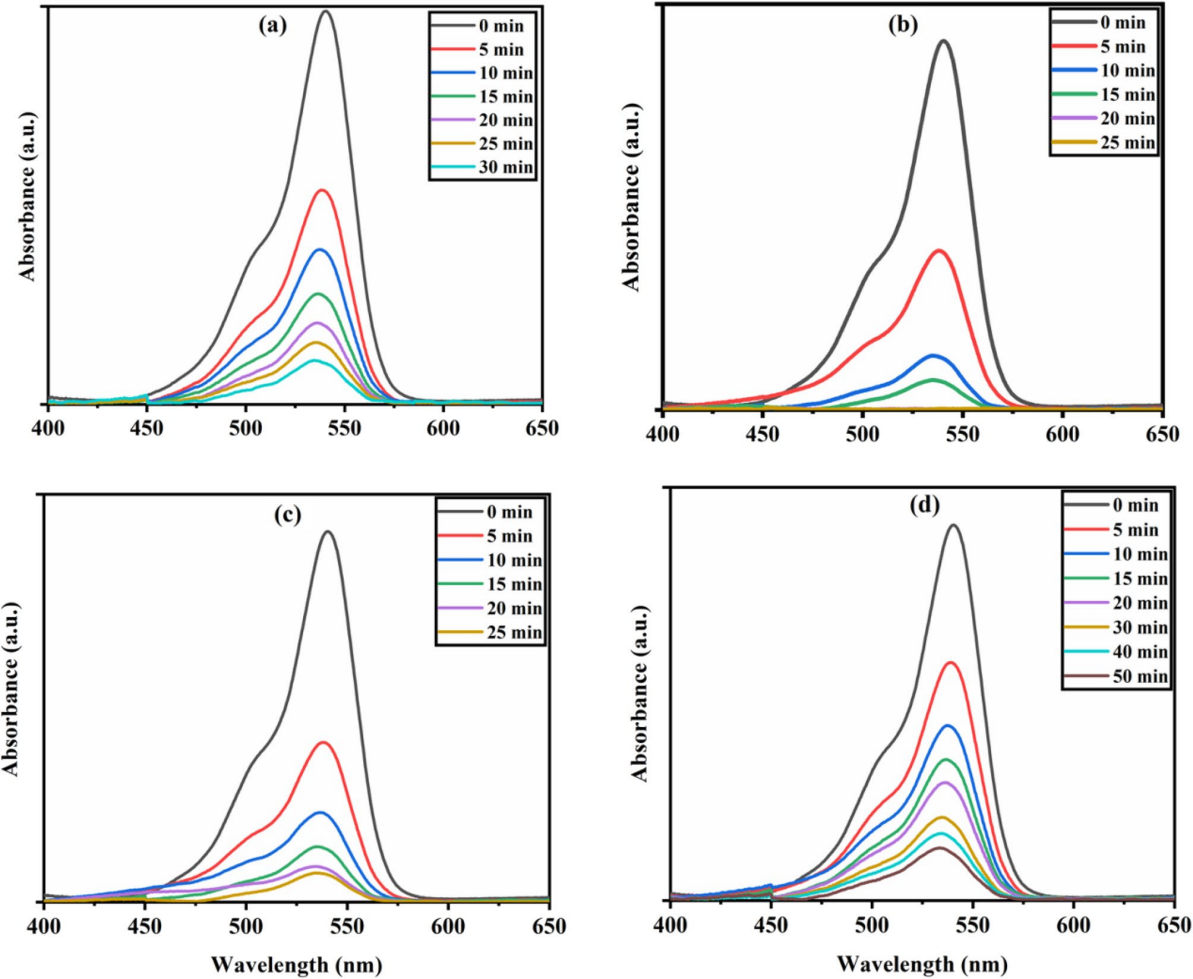
Photocatalytic degradation of (**a**) 5CNT (**b**) 10CNT (**c**) 15CNT (**d**) 20CNT composites against as prepared 10 ppm RB dye solution under visible light irradiation.

**Figure 7:**
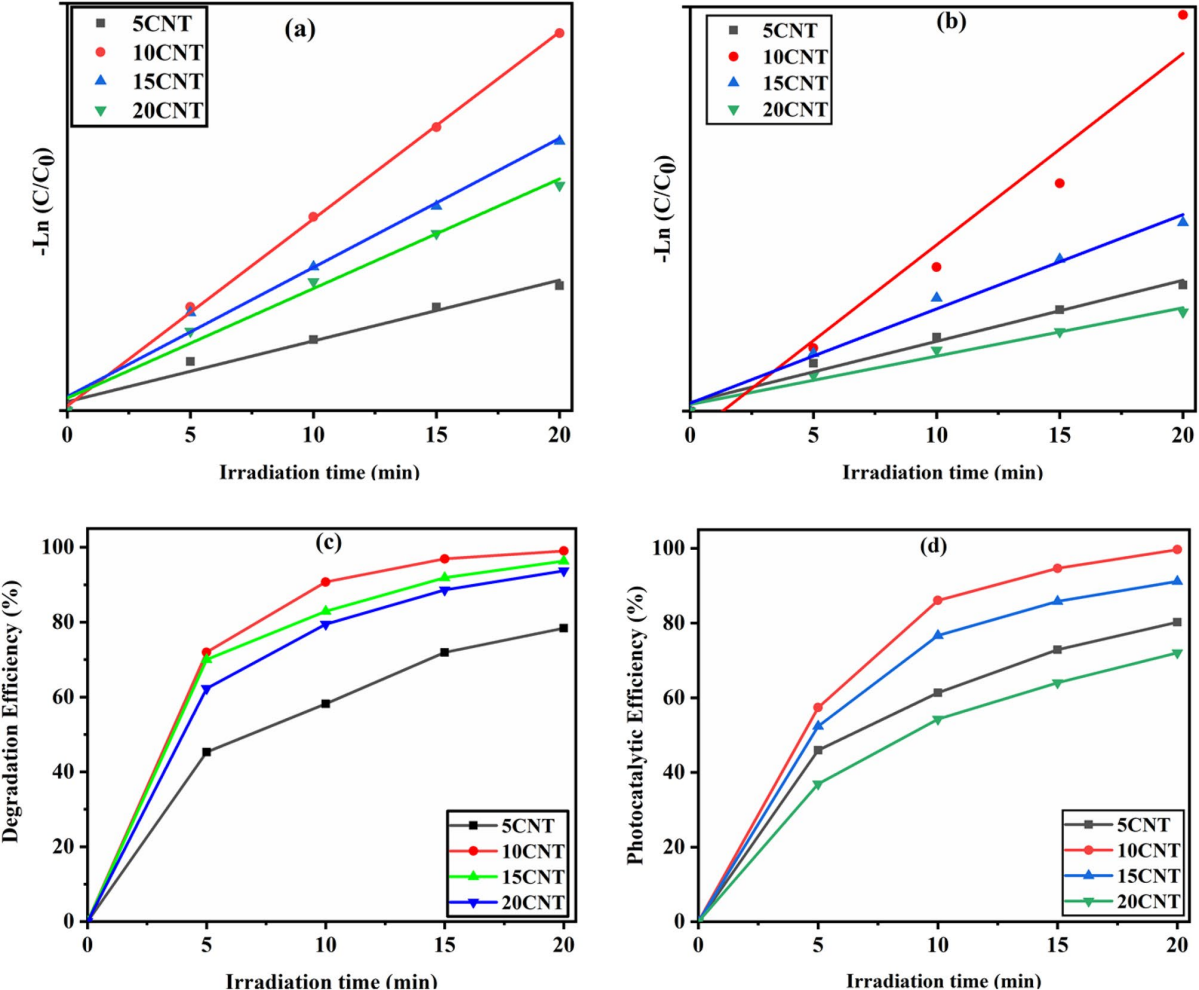
Graph of –Ln(C/C_0_) versus irradiation time for 10 ppm (**a**) MB dye, (**b**) RB dye, and photocatalytic degradation efficiency against irradiation time for 10 ppm (**c**) MB dye (**d**) RB dye.

The photocatalytic activity of the synthesized nanohybrid catalysts was initially studied in the presence of visible light with 10 ppm methylene blue (MB) and 10 ppm rose bengal (RB) as symbolic pollutants, as shown in Figs. 5 and 6. In the current study, we prepared an array of stock solutions of MB and RB dyes in 1000 ml DDW and stored them in the dark. Catalysts 5CNT, 10CNT, 15CNT, and 20CNT have shown dye disintegration efficiencies of 78%, 99%, 98%, and 93% within 20 min of visible light irradiation against 10 ppm MB dye solution, as shown in Fig. 7a and c. Similarly, 80%, 99%, 91%, and 74% dye degradation efficiency within 20 min of visible light irradiation against 10 ppm RB dye solution, respectively, as shown in Fig. 7b and d. The photocatalytic degradation efficiency was calculated using Eq. (3)^63^.

3 $${\text{Photocatalytic}}\text{ degradation efficiency}=\frac{{\text{C}}_{0}-{\text{C}}_{\text{t}}}{{\text{C}}_{\text{t}}}\text{ x }100$$

where C_0_ is the absorbance at time’ t = 0 min and C_t_ is the absorbance at time ‘t’ min.

Thus, the 10CNT catalysts showed optimized results for the disintegration of all dyes. The stability of 10CNT photocatalysts was studied by repeating the degradation studies, which have prolifically shown 91% efficiency even after four reusability cycles. The variation in the pH value of the dye solution was also explored during this study for the 10 ppm MB dye solution, and the photocatalytic degradation experiment was performed with pH values ranging from 6 to 10. We also carried out variations in catalytic doses from 0.6 g/liter, 0.8 g/liter, 1 g/liter, and 1.2 g/liter loading of photocatalysts against 10 ppm MB dye solutions at an optimum pH value-9 in the presence of visible-light irradiation. A spectroscopic study of fixed time interval samples was carried out using UV-Vis spectroscopy, which revealed that the preliminary absorption peak at 664 nm gradually declined with time. This indicates that the MB dye solution crumbled into constituents such as CO_2_, H_2_O, and other bland organic waste products^64^ (Table 2).

**Table 2:** Photocatalytic degradation parameters of 5CNT, 10CNT, 15CNT, and 20CNT catalysts under visible light irradiation for as prepared 10 ppm MB and RB dyes.

Sample	Rate constant (10^−2^) (min^−1^)		Half-life (min)		Degradation efficiency (%)	
	MB	RB	MB	RB	MB	RB
5CNT	11	9	6	7.5	88.15 ± 1.55	80.2 ± 1.61
10CNT	23	20	2.9	3.3	99.12 ± 1.50	99.7 ± 1.75
15CNT	21	13	3.2	5.2	98.77 ± 1.75	91.1 ± 1.63
20CNT	15	9	4.6	7.3	93.69 ± 1.83	72.2 ± 1.75

The proposed mechanism of the electron–hole pair generation and photocatalytic degradation by Ag doped ZnO:CNT is as shown below^29,30^.

4 $${\text{Ag}} + {\text{hv}}\left( {{\text{visible}}\;{\text{light}}} \right) \to {\text{h}}^{ + } + {\text{e}}^{ - }$$

5 $${\text{Ag}}\left( {{\text{e}}^{ - } } \right) + {\text{ZnO}} \to {\text{ZnO}}\left( {{\text{e}}_{{{\text{CB}}}}^{ - } } \right) + {\text{Ag}}\left( {{\text{h}}^{ + } } \right)$$

6 $${\text{ZnO}}\left( {{\text{e}}^{ - } } \right) + {\text{CNT}} \to {\text{CNT}}\left( {{\text{e}}_{{{\text{CB}}}}^{ - } } \right) + {\text{ZnO}}\left( {{\text{h}}^{ + } } \right)$$

7 $${\text{CNT}}\left( {{\text{e}}_{{{\text{CB}}}}^{ - } } \right) + {\text{O}}_{{2}} \to {\text{CNT}} + {\text{O}}_{{2}}^{ \bullet - }$$

8 $${\text{H}}_{{2}} {\text{O}} \to^{ - } {\text{OH}} + {\text{H}}^{ + }$$

9 $${\text{O}}_{{2}}^{ \bullet - } + {\text{H}}^{ + } \to {\text{2HO}}_{{2}}^{ \bullet } \to {\text{H}}_{{2}} {\text{O}}_{{2}} \to^{ \bullet } {\text{OH}} + {\text{OH}} - + {\text{O}}_{{2}}$$

10 $${\text{ZnO}}\left( {{\text{h}}^{ + } } \right) + {\text{OH}}^{ - } \to {\text{ZnO}} +^{ \bullet } {\text{OH}}$$

11 $${\text{Dye}} +^{ \bullet } {\text{OH}} \to {\text{H}}_{{2}} {\text{O}} + {\text{Dye}}^{ + \bullet } \to {\text{degradation}}\;{\text{products}}$$

### Parameter variation in photocatalytic studies

**Figure 8:**
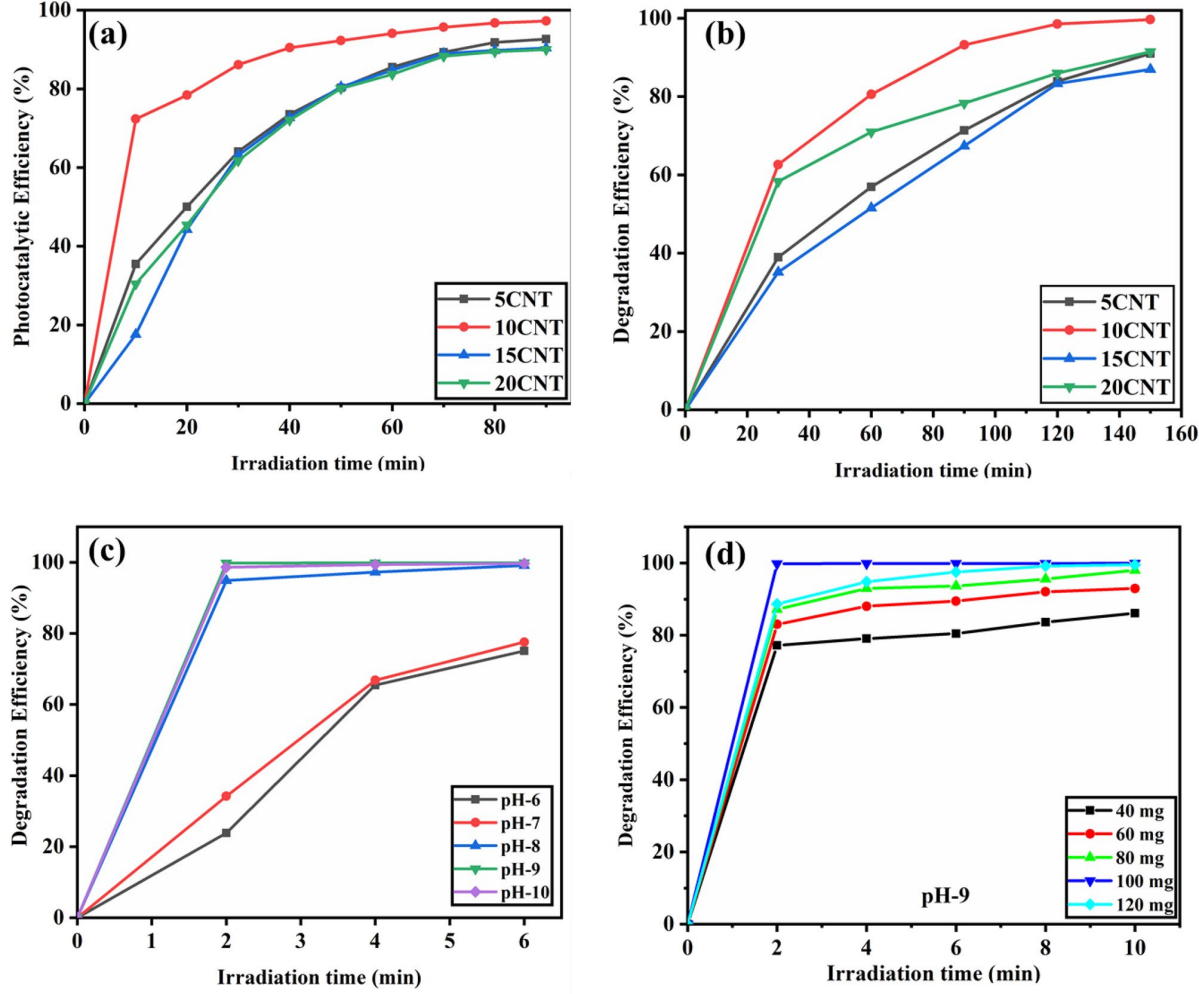
Photocatalytic degradation efficiency Vs. irradiation time of MB dye (**a**) at 30 ppm concentration (**b**) at 50 ppm concentration (**c**) pH variation (**d**) catalytic dose variation.

In this study, we explored aspects such as variations in dye concentration, pH, and catalytic dose to optimize these parameters. The dye concentration was optimized by studying 10, 30, and 50 ppm solutions of MB dye against 5CNT, 10CNT, 15CNT, and 20CNT catalysts. It was observed that with an increase in the concentration of the dye, there was an upsurge in the time required for photocatalytic disintegration, as shown in Fig. 8a,b, with significant variances as matched with 30 ppm and 50 ppm solutions. Thus, the dye concentration is inversely proportional to the dye degradation efficiency of the catalyst. The catalyst concentration inhibited dye degradation. The 10CNT catalysts have shown 99% degradation efficiency at an MB dye concentration of 10 ppm. Based on this information, we tried varying the pH and catalytic dosage of the 10 ppm MB dye solution against 10CNT catalysts.

The consequence of the variation in the pH of 10 ppm MB dye solution on the disintegration performance was calculated using 100 mg of 10CNT catalyst, as displayed in Fig. 8c. The initial pH of the MB solution was ~ 6.9. With an increase in pH, there was considerable reduction in photocatalytic degradation time Here, the pH for MB was varied from 6 to 10, showing the best result at pH 9, with 100% degradation within 2 min of visible light irradiation^65^.

Hence, we performed photocatalytic degradation of MB dyes using four different weight ratios of the 10CNT catalysts, that is, 0.6, 0.8, 1, and 1.2 g g/L at a modified pH of 9^36^. As shown in Fig. 8d, the MB dye gave the best results at 1 g/liter for 10CNT. In this study, we observed that an increased catalytic dose was responsible for the improved degradation efficiency because it provided additional reaction locations for the dye molecules^66^. This effect may be caused by the snooping of the radiation flowing into the reaction mixture, which is attributed to the upsurge in the catalyst dose^67^. This might also be due to the inflated quantity of hydroxyl and superoxide radicals produced by increasing the catalyst mass. Beyond the 1 g/L catalytic dose, no significant change in the photocatalytic dye degradation performance was observed.

Repeated use of the 10CNT catalysts was tested by accumulating the catalyst mass after the dye disintegration reaction was completed. The samples were cleaned and filtered multiple times using ethanol and DDW. To verify the reusability of the collected samples, they were dried and used again under the same experimental conditions. Thus the 10CNT sample sustained up to four cycles, showing 91%efficiency against MB dye even at the end of the fourth cycle.

### Antifungal activity

**Figure 9:**
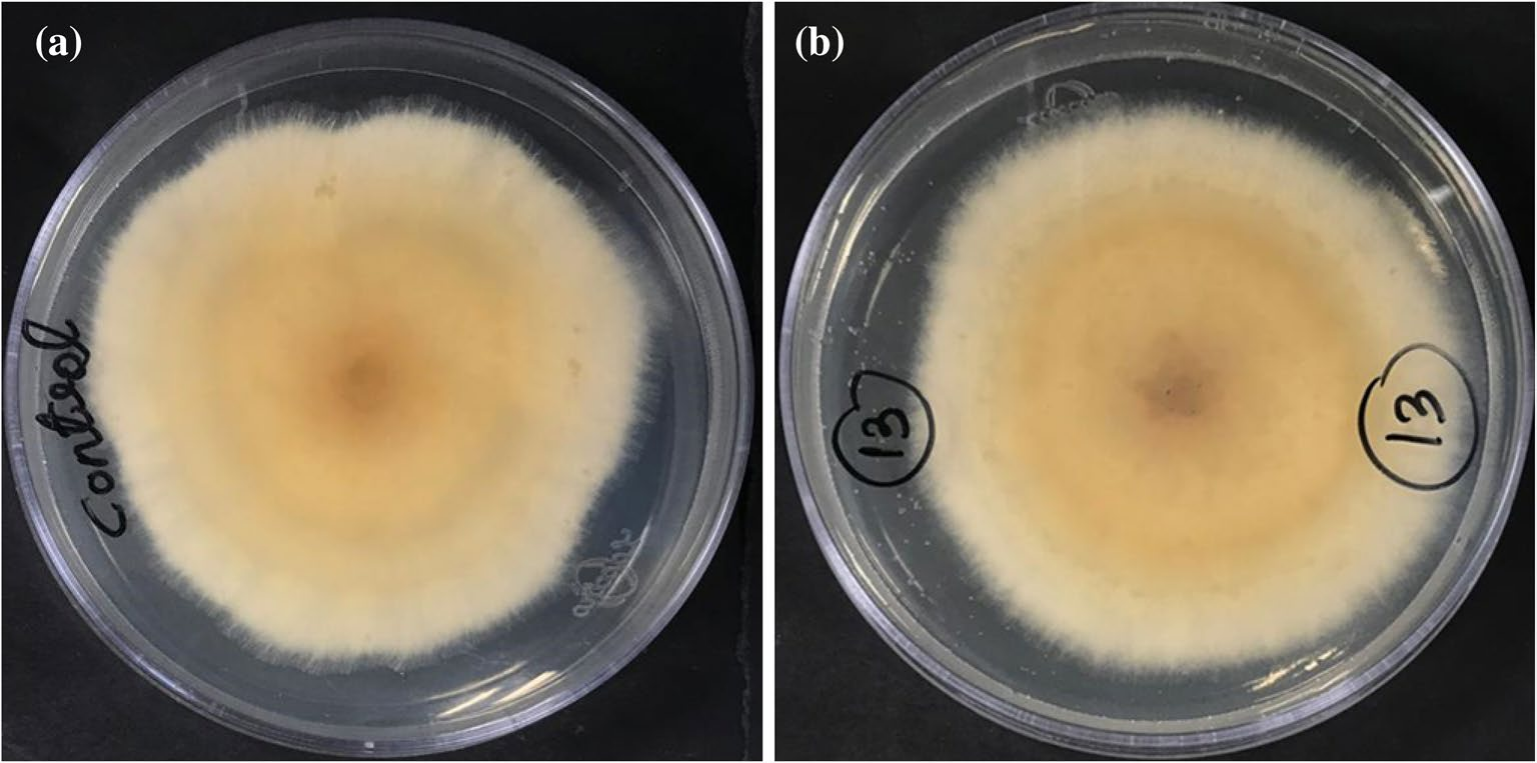
Antifungal activity of Ag-loaded ZnO:CNT against *Bipolaris sorokiniana* for (**a**) control (**b**) 5CNT.

In this study, we evaluated the antifungal activity of the phytopathogenic fungus *Bipolaris sorokiniana*, which causes spot blotch infections in wheat crops. Potato dextrose agar (PDA) is a regularly used medium for fungal culture, making it an appropriate substrate for testing the antifungal activity of catalysts. PDA is composed of potato infusion and dextrose, both of which supply nutrients for fungal development. The 5CNT, 10CNT, 15CNT, and 20CNT catalysts were added to the media at a specified concentration to test the antifungal activity. The medium was subsequently injected with fungal spores and fungal development was tracked over time. The antifungal activity of the substance under test is determined by the suppression of fungal growth, which manifests as a lack of apparent growth or a reduction in the number of fungal colonies. These findings suggest that Ag-loaded ZnO has substantial potential as a substitute for synthetic fungicides in the management of plant diseases^36^. The photo-induced generation of ROS and the poisoning effect due to Zn^2+^ release are the two key contributors to the antifungal performance of ZnO nanoparticles. The mechanism of action of Ag-loaded ZnO:CNT against fungi involves the creation and accumulation of ROS and free radicals, which primarily disturb the cell wall, surface proteins, and nucleic acids of the fungi. In addition, they block proton pumps. This suggests that Ag-loaded ZnO:CNT has the potential to be an effective treatment for fungal infections^68^. The antifungal activity of Ag-loaded ZnO:CNT was evaluated using the poisoned food method, as shown in Fig. 9^69^. The diameter was measured using the Kirby-Bauer method, as shown in Eq. (12).

12 $$\% Antifungal Activity=\frac{({D}_{c}-{D}_{S})}{{D}_{c}} \times 100$$

where D_c_ is the diameter of growth in the control plate and D_s_ is the diameter of the plate containing Ag-loaded ZnO:CNT nanoparticles. The % antifungal activities of the 5CNT, 10CNT, 15CNT, and 20CNT were 48%, 46%, 43%, and 40%, respectively.

### Antibacterial activity

The Ag-loaded ZnO:CNT composites were evaluated for their antimicrobial activity against *Staphylococcus aureus* (NCIM-2654), *Bacillus cerius* (NCIM-2703), *Proteus vulgaris* (NCIM-2813), and *Salmonella typhimurium* (NCIM-2501). The inoculum for microbial cultures was produced in sterile saline water. Bacterial growth was conducted on nutrient agar plates. Cultures of *S. aureus*, *B. cerius*, *P. vulgaris*, and *S. typhimurium* were spread out on a sterile nutrient agar plate. Sterile corkborers measuring 5 mm were used to create wells on these plates. Using a micropipette, 100 µg/ml of the synthesized substance was distributed in sterile dimethyl sulfoxide (DMSO). To evaluate antibacterial activity, the plates were incubated for 24 h at 370 °C. The antibacterial activity of the composite material was examined in conjunction with a blank dimethyl sulfoxide (DMSO) negative control. Using streptomycin as the reference medication, the antibacterial abilities of the composite materials were investigated using the agar well gel diffusion method. This study demonstrates the considerable antibacterial performance of the prepared composites because they curtail the growth of the given bacterial strains. Initially, DMSO medium was prepared using a liquid auger suspension. Bacterial strains were prepared separately for each sample and control, and the bacterial control suspensions were incubated for 12 h. at 37 °C^70,71^.

**Figure 10:**
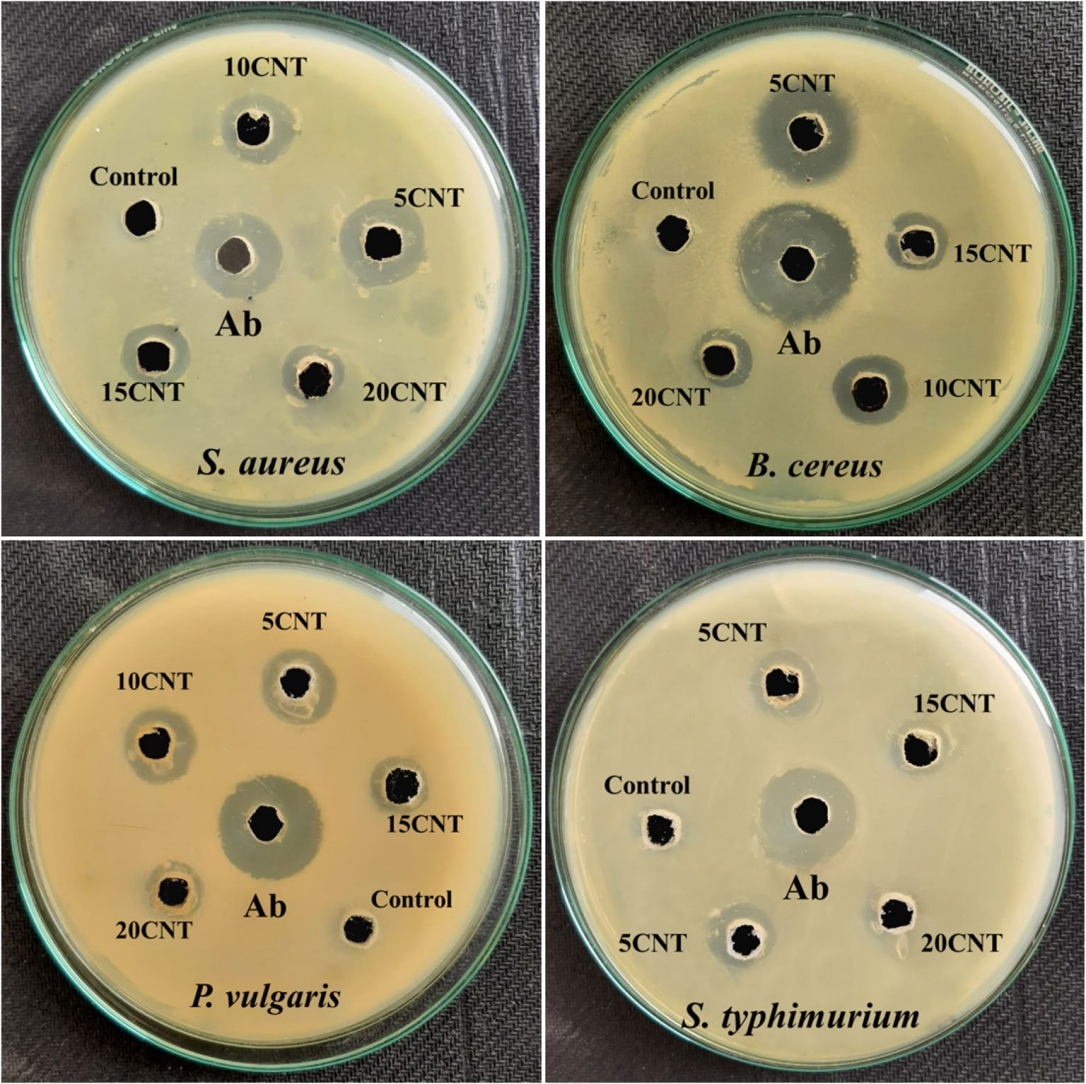
Zone of inhibition: of 5CNT, 10CNT, 15CNT, 20CNT and an antibiotic against *S. aureus*, *B. cereus*, *P. vulgaris*, and *S. typhimurium*.

The Ag-loaded ZnO:CNT composite samples were prepared at 2.5 mg/mL, and 10 µL drops of the sample were mixed with the above bacterial suspensions. Figure 10 shows the antibacterial activity of Ag-loaded ZnO:CNT catalysts against the bacterial strains, using streptomycin as a positive control and dimethyl sulfoxide (DMSO) and distilled water as a negative control. In the present study, the zone of inhibition (ZOI) values revealed that the 5CNT composite was more effective in constraining the development of the bacterial strain *B. cerius*, as the maximum ZOI was 21.66 ± 0.57 mm. The ZOIs of 5CNT composite against *S. aureus*, *P. vulgaris*, and *S. typhimurium* bacterial strains were 17.68 ± 0.57 mm 15.66 ± 0.57 mm, and 13.66 ± 0.57 mm, respectively, as illustrated in Table 3. The commercial drug streptomycin, which is exclusively effective against specific bacterial strains, was used to compare the outcomes. The ZOIs were 17.66 ± 0.57 mm, 25.66 ± 0.57 mm, 20.33 ± 0.57 mm, and 19.00 ± 1.00 mm for *S. aureus*, *B. cerius*, *P. vulgaris*, and *S. typhimurium*, respectively^72^. The aforementioned information shows the average ± standard error of three replicates (Table 3).

**Table 3:** The zone of inhibition of bacterial strains *S. aureus*, *B. cerius*, *P. vulgaris*, and *S. typhimurium*.

Catalyst	Antibacterial activity			
	Gram-positive		Gram-negative	
	*Staphylococcus aureus* (NCIM-2654)	*Bacillus cereus* (NCIM-2703)	*Proteus velgaris* (NCIM-2813)	*Salmonella typhimurium* (NCIM-2501)
5CNT	17.68 ± 0.57	21.66 ± 0.57	15.66 ± 0.57	13.66 ± 0.57
10CNT	15.01 ± 1.00	16.00 ± 1.00	14.16 ± 0.57	11.33 ± 1.15
15CNT	14.00 ± 1.00	13.66 ± 0.57	11.66 ± 0.57	11.00 ± 1.00
20CNT	12.13 ± 0.23	11.66 ± 0.57	10.33 ± 0.57	08.66 ± 1.15
Antibiotic (Ab)	17.66 ± 0.57	25.66 ± 0.57	20.33 ± 0.57	19.00 ± 1.00

## Conclusions

In this study, Ag-loaded ZnO:CNT nanocomposites were prepared using a refluxed chemical method. XRD analysis confirmed the structural clarity of the Ag-loaded ZnO:CNT nanocomposites. XRD investigation revealed the presence of silver and carbon peaks, and the average crystallite size ranged from 19 to 25 nm. The microstrain and dislocation density for Ag-loaded ZnO:CNT changed slightly with a change in the CNT content. The lattice parameters, crystal structure, and band gap energy of the Ag-loaded ZnO:CNT crystal structures for 5CNT, 10CNT, 15CNT, and 20CNT were found to be very similar. FE-SEM and EDS analyses confirmed the presence of Ag-loaded ZnO on the exterior of the CNT. The FESEM micrograph shows an agglomerated nano-flower-like morphology. The photocatalytic activities of MB and RB dyes were higher for the 10CNT. There was an increase in the photocatalytic efficiency with an increase in the pH of the dye solution up to the optimum value. The optimum result for MB dye was observed for 10CNT at pH 9, showing 100% photocatalytic efficiency in 2 min at a rate constant 1.48 min^−1^. While The RB dye exhibited 99% photocatalytic efficiency in 20 min at pH 5 for 10CNT at a rate of 0.20 min^−1^. The reuse of the 5CNT composite has shown excellent sustainability, it has shown 91% efficiency after 4th cycle. The variation in the MB dye concentration from 10 to 50 ppm revealed that the time required to degrade the dye solution increased with increasing dye concentration. In addition, we investigated the use of Ag-loaded ZnO:CNT as an antifungal agent against *Bipolaris sorokiniana*. It was observed that 5CNT showed better inhibition of *Bipolaris sorokiniana* fungus showing 48% efficiency. The 5CNT samples were tested against bacterial strains *Staphylococcus aureus*, *Bacillus cerius*, *Proteus vulgaris*, and *Salmonella typhimurium* and showed promising antibacterial activity in comparison with commercially available drugs.

In future research, we plan to incorporate advanced characterization techniques such as Electrochemical Impedance Spectroscopy (EIS) and Transmission Electron Microscopy (TEM) to gain deeper insights into the charge transfer dynamics and detailed structural properties of our silver stacked zinc oxide garnished on carbon nanotube composites. These analyses will complement our current Field Emission Scanning Electron Microscopy (FESEM) studies and provide a more comprehensive understanding of the materials' photocatalytic, antibacterial, and antifungal activities.

## Data Availability

The datasets generated or analyzed during the current study are available from the corresponding author upon reasonable request.
